# Identification of reactive intermediate formation and bioactivation pathways in Abemaciclib metabolism by LC–MS/MS: *in vitro* metabolic investigation

**DOI:** 10.1098/rsos.181714

**Published:** 2019-01-23

**Authors:** Adnan A. Kadi, Hany W. Darwish, Hatem A. Abuelizz, Thamer A. Alsubi, Mohamed W. Attwa

**Affiliations:** 1 Department of Pharmaceutical Chemistry, College of Pharmacy, King Saud University, PO Box 2457, Riyadh 11451, Saudi Arabia; 2 Analytical Chemistry Department, Faculty of Pharmacy, Cairo University, Kasr El-Aini Street, Cairo 11562, Egypt

**Keywords:** Abemaciclib, reactive metabolites, iminium intermediates, side effects

## Abstract

Abemaciclib (Verzenio^®^) is approved as a tyrosine kinase inhibitor (TKI) for breast cancer treatment. In this study, *in vitro* phase I metabolic profiling of Abemaciclib (ABC) was done using rat liver microsomes (RLMs). We checked the formation of reactive intermediates in ABC metabolism using RLMs in the presence of potassium cyanide (KCN) that was used as a capturing agent for iminium reactive intermediates forming a stable complex that can be characterized by LC–MS/MS. Nine *in vitro* phase I metabolites and three cyano adducts were identified. The metabolic reactions involved in the formation of these metabolites and adducts are reduction, oxidation, hydroxylation and cyanide addition. The bioactivation pathway was also proposed. Knowing the electrodeficient bioactive centre in ABC structure helped in making targeted modifications to improve its safety and retain its efficacy. Blocking or isosteric replacement of α-carbon to the tertiary nitrogen atoms of piperazine ring can aid in reducing toxic side effects of ABC. No previous articles were found about *in vitro* metabolic profiling for ABC or structural identification of the formed reactive metabolites for ABC.

## 1. Introduction

A malignant tumour is formed of a group of cancer cells that have the ability to grow and invade tissues either surrounding or at distant areas of the body [1]. Globally, breast cancer is the most frequently diagnosed cancer in women; it affects about 12% of women worldwide [2]. Saudi Arabia is no exception, where breast cancer is most commonly prevalent. It accounts for about 22% of all new cancers in women that are ranked first figures [3]. There is a group of novel drugs that specifically target gene changes in cancer cells that help the cells grow or spread. Tyrosine kinases (TKs) are important targets because of their important role in the modulation of growth factor signalling [4]. Controlling the activity of TK in the cell regulates many vital processes such as cell cycle, proliferation and cell death [5].

**Figure 1.**
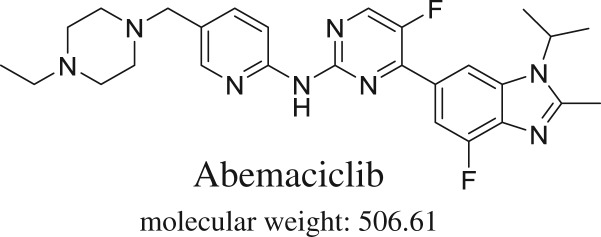
ABC chemical structure.

Cyclin-dependent kinases 4 and 6 (CDK4/6), as a class of tyrosine kinase inhibitor (TKI), play a crucial role in cell proliferation. When CDK4/6 pathway is dysregulated, it leads to an implication in breast cancer biology [6]. There are three highly selective CDK4/6 inhibitors that have been approved for breast cancer therapy: Palbociclib, Ribociclib and Abemaciclib (ABC) (Verzenio^®^) [7]. ABC (figure 1) is the third agent in this class, which was approved in September 2017 by the Food and Drug Administration (FDA), either in combination with fulvestrant for women or as monotherapy for women and men with HR-positive, HER2-negative advanced or metastatic breast cancer. Lately, in February 2018, FDA approved ABC with an aromatase inhibitor as the first-line endocrine-based therapy for metastatic breast cancer. The most frequent side effects of the drug were diarrhoea, which occurred in approximately 80% of patients, neutropenia, fatigue, infections, nausea, abdominal pain, anaemia, vomiting, alopecia, decreased appetite and leukopenia [8].

Our group previously studied reactive metabolite formations of some TKI drugs. The current work proved that ABC formed three reactive intermediates through a specific bioactivation pathway. KCN was used as a trapping agent to capture the formed reactive metabolites and the bioactivation pathways were characterized [8–12]. The chemical structures of ABC contain *N*-ethyl piperazine ring. This group is expected to undergo metabolism generating reactive iminium intermediates that can be trapped by KCN forming cyano adducts. Oxidation at a carbon alpha to an N-substituted piperazine ring is known to result in iminium ions, which are considered hard electrophiles and can be trapped by cyanide anions to form cyano adducts. The N-substituted piperazine partial structures become bioactivated and subsequently trapped by the nucleophilic cyanide anion. The reactive intermediates were postulated as imine and imine-carbonyl conjugate (α,β-unsaturated) structures on the piperazine ring. Bioactivation of the N-substituted piperazine partial structure, which can be characterized and detected using tandem mass spectrometry (LC–MS/MS), is a possible explanation for reported idiosyncratic toxicity [13–16].

Iminium intermediates initiate several toxic side effects through previously reported mechanisms as in the case of brigatinib and ponatinib that bind covalently to a DNA base [9,17]. Knowing the electrodeficient bioactive centre in ABC structure helped in making targeted modifications to improve its safety and retain its efficacy. ABC rat liver microsome (RLM) incubation resulted in the identification of nine *in vitro* phase I metabolites and three cyano conjugates, and the proposed reactions involved include reduction, oxidation, hydroxylation and cyanide addition.

## 2. Chemicals and methods

### 2.1. Chemicals

All chemicals are described in table 1. RLMs were made in house using Sprague-Dawley rats following previously reported methods [9,17–19]. The experimental design for animal work was approved by the University's Ethics Review Committee.

**Table 1.** List of chemicals and materials.

name	source
Abemaciclib	Med. Chem. Express (NJ, USA)
acetonitrile (HPLC grade), ammonium formate, potassium cyanide and formic acid	Sigma-Aldrich (USA)
water (HPLC grade)	Milli-Q plus filtration system (USA)
rats (Sprague-Dawley)	King Saud University experimental animal care center (Saudi Arabia)

### 2.2. Chromatographic conditions

An Agilent 6410 QQQ equipped with an ESI coupled to an Agilent 1200 HPLC was used. The liquid and mass chromatographic parameters were adjusted for each drug. ABC and its metabolites were produced in the collision cell by CID. The optimized conditions for chromatographic resolution of incubation mixture extract are given in table 2.

**Table 2.** Chromatographic parameters of the proposed LC–MS/MS methodology.

liquid chromatography			mass spectrometry	
RRLC	Agilent 1200 series		mass spectrometer	Agilent 6410 QQQ
mobile phase	A: 0.1% formic acid		ESI source	positive ESI
	B: ACN			N_2_ of low purity as drying gas: At flow rate of 12 l min^−1^ and 60 psi pressure
	flow rate: 0.25 ml min^−1^			
	run time: 75 min			
Agilent Zorbax eclipse plus C_18_ column	L	150 mm		ESI temperature: 350°C
	ID	2.1 mm		capillary voltage: 4000 V
	PS	3.5 µm	gas inside collision cell	N_2_ of high purity
	T	22 ± 1°C	detection modes	MS scan and PI
elution system	time in minutes	% ACN	drug	ABC and its *in vitro* and reactive metabolites
	0	5		
	5	5	fragmentation	fragmentor voltage (FV): 135 V
	30	60		collision energy (CE): 20 eV
	40	90		
	45	5		

### 2.3. RLM incubations

ABC was incubated at 20 µM with 1 mg ml^−1^ RLMs, 1 mM NADPH, 1 mM KCN and 50 mM Na/K phosphate buffer (pH 7.4) containing 3.3 mM MgCl_2_. The mixtures were incubated at 37°C in a shaking water bath for 60 min before the metabolic reactions were terminated using protein precipitation by adding 2 ml of ice-cold ACN followed by centrifugation at 9000 g for 10 min at 4°C. The supernatants were removed to clean vials then evaporated to dryness, reconstituted in the mobile phase and analysed by the LC/MS system [8,12,17,20]. Two controls were done in the absence of NADPH or RLMs to confirm that ABC phase I metabolites were metabolically formed.

### 2.4. Characterization of ABC reactive intermediates in *in vitro* metabolic reactions

The same RLM metabolic incubation with ABC, previously described in §2.3, was repeated but in addition to 1.0 mM KCN to trap reactive iminium intermediates. This experiment was repeated three times to confirm the results and support our conclusions. Two controls were done in the absence of NADPH or KCN to confirm that cyano adducts are formed due to metabolic bioactivation.

### 2.5. Identification of ABC reactive metabolites

MS scan and extracted ion chromatogram (EIC) detection modes were used to characterize and locate metabolites in the incubation mixtures, while product ion (PI) was used to identify ABC *in vitro* metabolites and adducts of reactive intermediates formed in ABC metabolism. Locating metabolites in metabolic mixture extract chromatogram was performed by EIC of *m/z* of the supposed ABC metabolites.

## 3. Results and discussion

### 3.1. PI study of ABC

**Figure 2.**
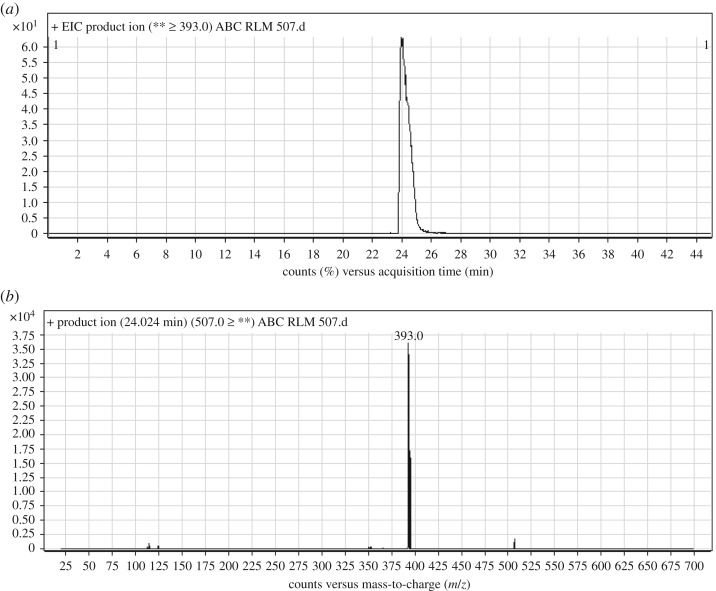
ABC PI chromatogram (*a*) and ABC PI MS spectrum (*b*).

**Scheme 1.**
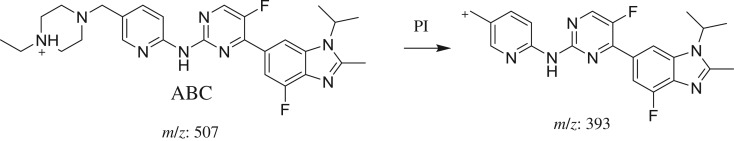
PIs of ABC.

ABC chromatographic peak appears at 24.0 min in PI chromatogram (figure 2*a*). Collision-induced dissociation (CID) of ABC at *m/z* 507 generates one fragment ion (FI) at *m/z* 399 by the loss of ethyl piperazine moiety (figure 2*b* and scheme 1).

### 3.2 Identification of *in vitro* and reactive ABC metabolites

After the purification and extraction of ABC RLM incubations, 15 µl was injected into LC–MS/MS. ABC incubation led to the identification of nine *in vitro* phase I metabolites and three cyano adducts, and the proposed reactions involved include reduction, oxidation, hydroxylation and cyanide addition (table 3). All these metabolites were formed in all incubations when repeated three times; this confirmed the validity of the used method. These results were confirmed by the absence of identified phase I metabolites and cyano adducts in all controls.

**Table 3.** Identified *in vitro* and cyano adducts of ABC.

	MS scan	MS^2^ product ions	*t*_R_ (min)	proposed phase 1 metabolic reaction
ABC	507	393	23.9	
*In vitro* ABC metabolism				
ABC493a	493	378.8	22.8	demethylation
ABC493b	493	378.9	25.8	demethylation
ABC509	509	395.1, 509	24.3	reduction
ABC521	521	365, 406.5, 521	28.8	methyl oxidation
ABC523a	523	393	23.2	ethyl hydroxylation
ABC523b	523	130, 393, 421, 495, 523	26.5	piperazine hydroxylation
ABC525a	525	395.1	23.32	hydroxylation and reduction
ABC525b	525	395.1	26.4	hydroxylation and reduction
ABC537	537	392.5	30.74	hydroxylation and oxidation
cyano adducts				
ABC532a	532	392.9	26.5	cyanide addition
ABC532b	532	393	37.2	cyanide addition
ABC548	548	393	29.36	hydroxylation and cyanide addition

#### 3.2.1. Identification of ABC493a and ABC493b phase I metabolites of ABC

**Figure 3.**
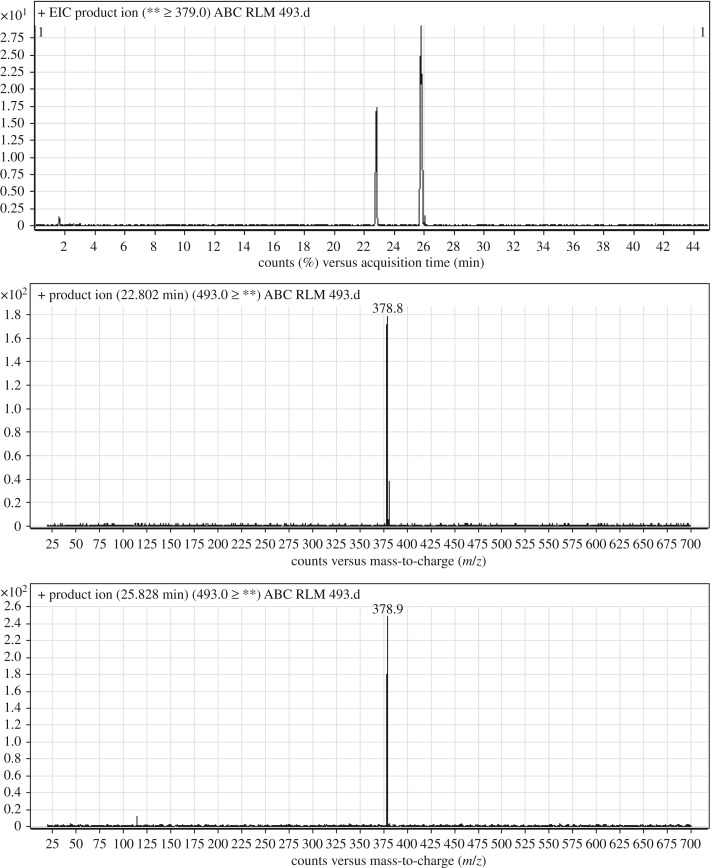
ABC493 PI chromatogram (*a*), ABC493a PI MS spectrum (*b*) and ABC493b PI MS spectrum (*c*).

**Scheme 2.**
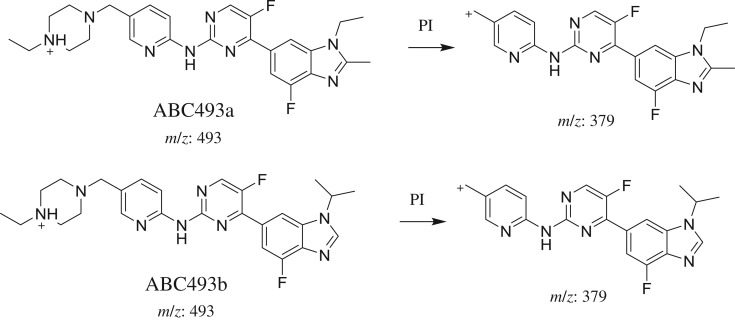
PIs of ABC493a and ABC493b.

ABC493a and ABC493b chromatographic peaks appear at 22.8 and 25.8 min, respectively, in PI chromatogram (figure 3*a*). CID of AB493 generates one FI at *m/z* 379 (figure 3*b*,*c*). Comparing with PIs of ABC, a decrease of 14 *m/z* units was identified, which indicates that demethylation metabolic reaction occurred in benzimidazole ring (scheme 2).

#### 3.2.2. Identification of ABC509 phase I metabolite of ABC

**Figure 4.**
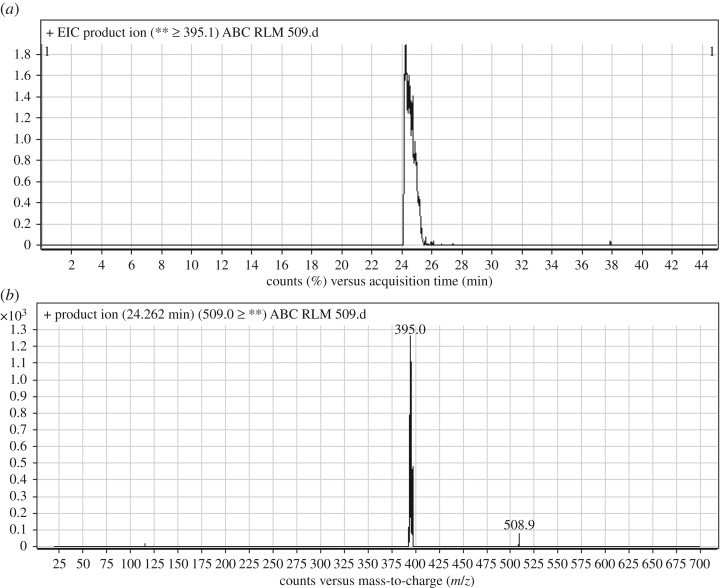
ABC509 PI chromatogram (*a*) and ABC509 PI MS spectrum (*b*).

**Scheme 3.**
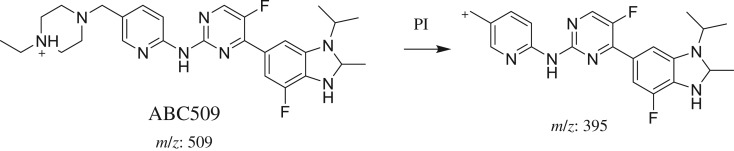
PIs of ABC509.

ABC509 chromatographic peak appears at 24.3 min in PI chromatogram (figure 4*a*). CID of ABC509 generates one FI at *m/z* 395 (figure 4*b*). Compared with PIs of ABC, an increase of 2 *m/z* units was identified, which indicates that reduction metabolic reaction occurred in benzimidazole ring (scheme 3).

#### 3.2.3. Identification of ABC521 phase I metabolite of ABC

**Figure 5.**
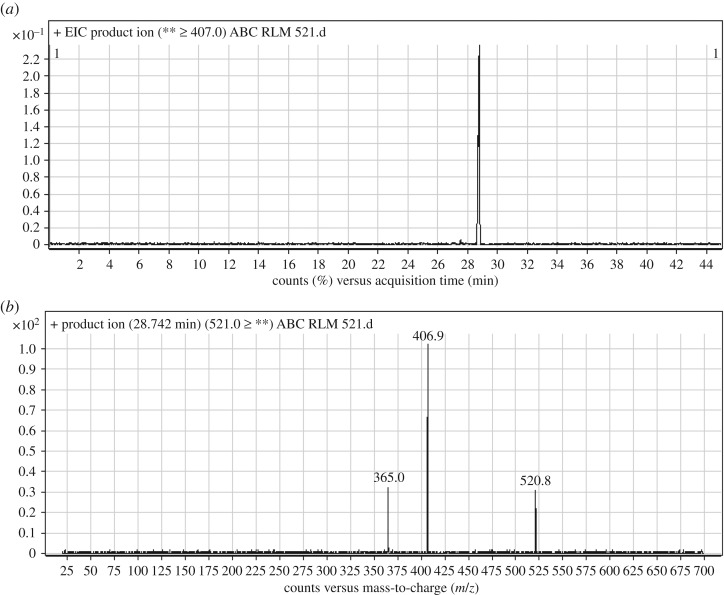
ABC521 PI chromatogram (*a*) and ABC521 PI MS spectrum (*b*).

**Scheme 4.**
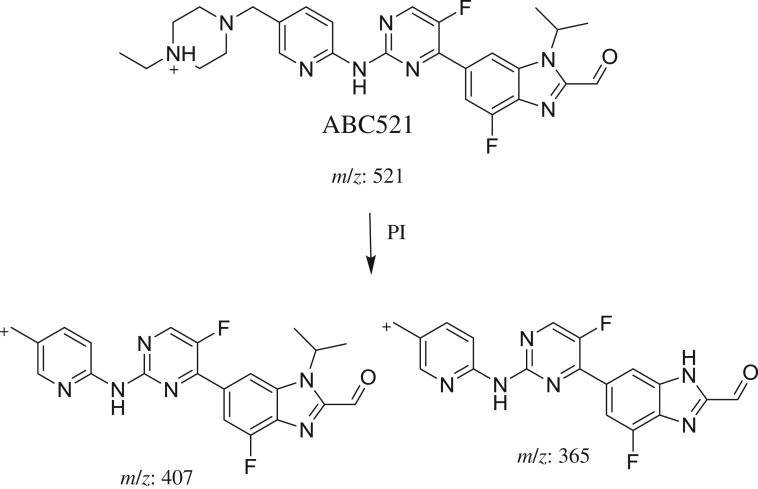
PIs of ABC521.

ABC521 chromatographic peak appears at 28.7 min in PI chromatogram (figure 5*a*). CID of ABC521 generates two FIs at *m/z* 407 and *m/z* 365 (figure 5*b*). Compared with PIs of ABC, an increase of 14 *m/z* units was identified, which indicates that oxidation metabolic reaction occurred in benzimidazole ring (scheme 4).

#### 3.2.4. Identification of ABC523a and ABC523b phase I metabolites of ABC

**Figure 6.**
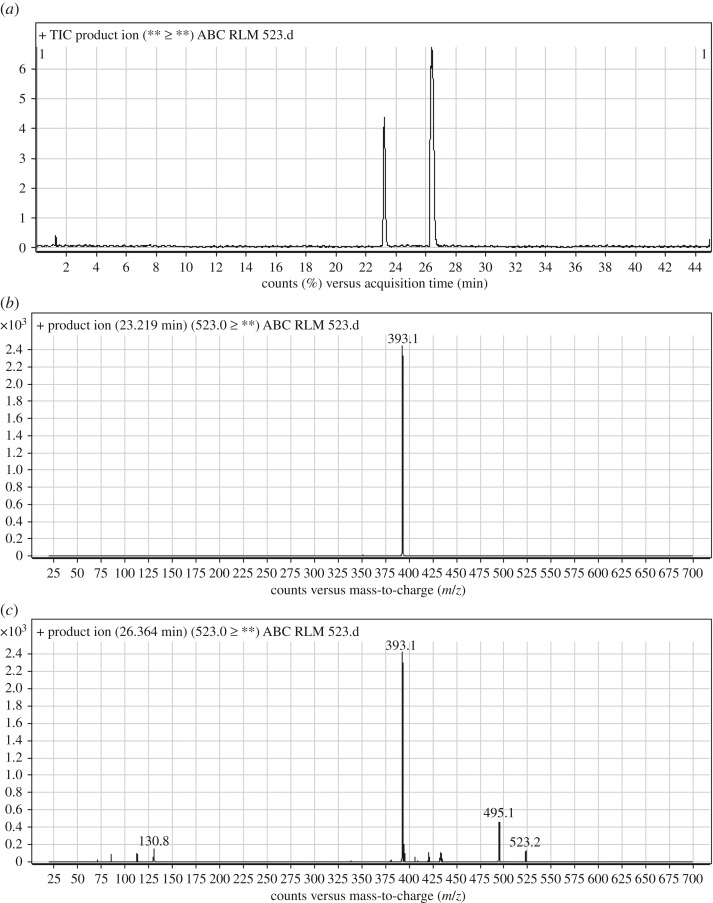
ABC523 PI chromatogram (*a*), ABC523a PI MS spectrum (*b*) and ABC523b PI MS spectrum (*c*).

**Scheme 5.**
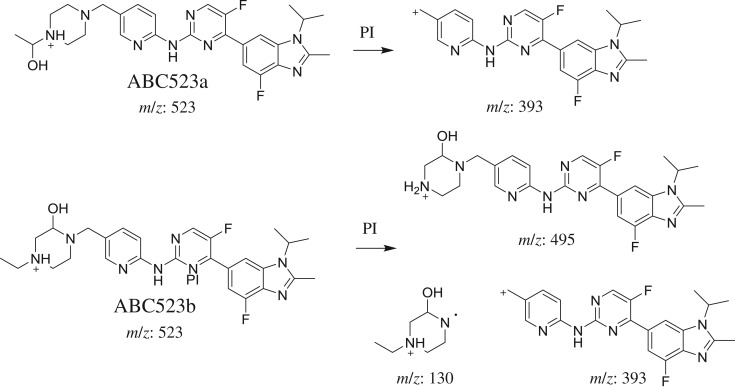
PIs of ABC523a and ABC523b.

ABC523a and ABC523b chromatographic peaks appeared at 23.2 and 26.4 min, respectively, in PI chromatogram (figure 6*a*). CID of ABC523a generates one FI at *m/z* 393 (figure 6*b*). CID of ABC523b generates three FIs at *m/z* 495, *m/z* 393 and *m/z* 130 (figure 6*c*). Compared with PIs of ABC, an increase of 16 *m/z* units was identified, which indicates that hydroxylation metabolic reaction occurred in *N*-ethyl piperazine ring (scheme 5).

#### 3.2.5. Identification of ABC525a and ABC525b phase I metabolites of ABC

**Figure 7.**
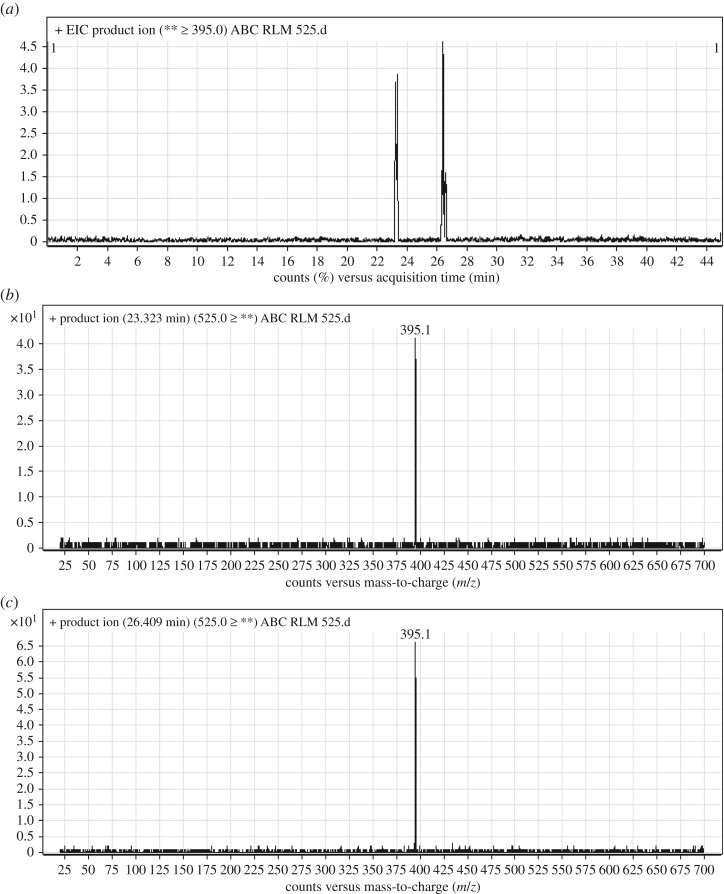
ABC525 PI chromatogram (*a*), ABC525a PI MS spectrum (*b*) and ABC525b PI MS spectrum (*c*).

**Scheme 6.**
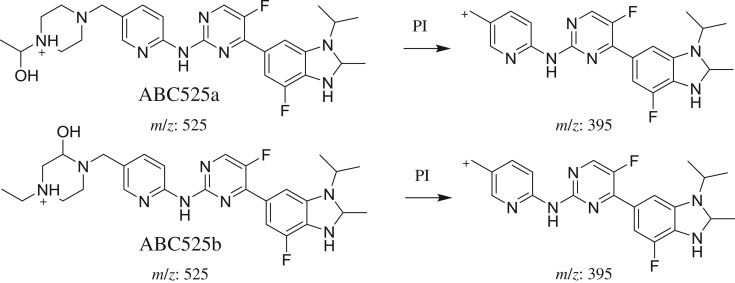
PIs of ABC525a and ABC525b.

ABC525a and ABC525b chromatographic peaks appeared at 23.3 and 26.4 min, respectively, in PI chromatogram (figure 7*a*). CID of ABC525 metabolites generates one FI at *m/z* 395 (figure 7*b*,*c*). Compared with PIs of ABC, an increase of 18 *m/z* units was identified, which indicates that reduction metabolic reaction occurred in benzimidazole ring in addition to hydroxylation metabolic reaction that occurred in *N*-ethyl piperazine ring (scheme 6).

#### 3.2.6. Identification of ABC537 phase I metabolite of ABC

**Figure 8.**
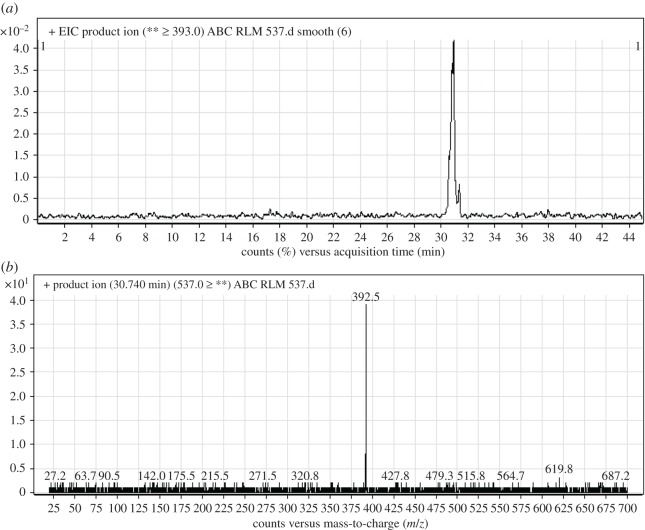
ABC537 PI chromatogram (*a*) and ABC537 PI MS spectrum (*b*).

**Scheme 7.**
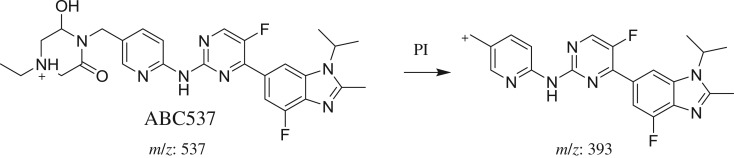
PIs of ABC537.

ABC537 chromatographic peak appears at 30.7 min in PI chromatogram (figure 8*a*). CID of ABC537 generates one FI at *m/z* 393 (figure 8*b*). Compared with PIs of ABC, an increase of 30 *m/z* units was identified, which indicates hydroxylation and oxidation metabolic reactions occurred in *N*-ethyl piperazine ring (scheme 7).

#### 3.2.7. Identification of ABC532a and ABC532b cyano adducts of ABC

**Figure 9.**
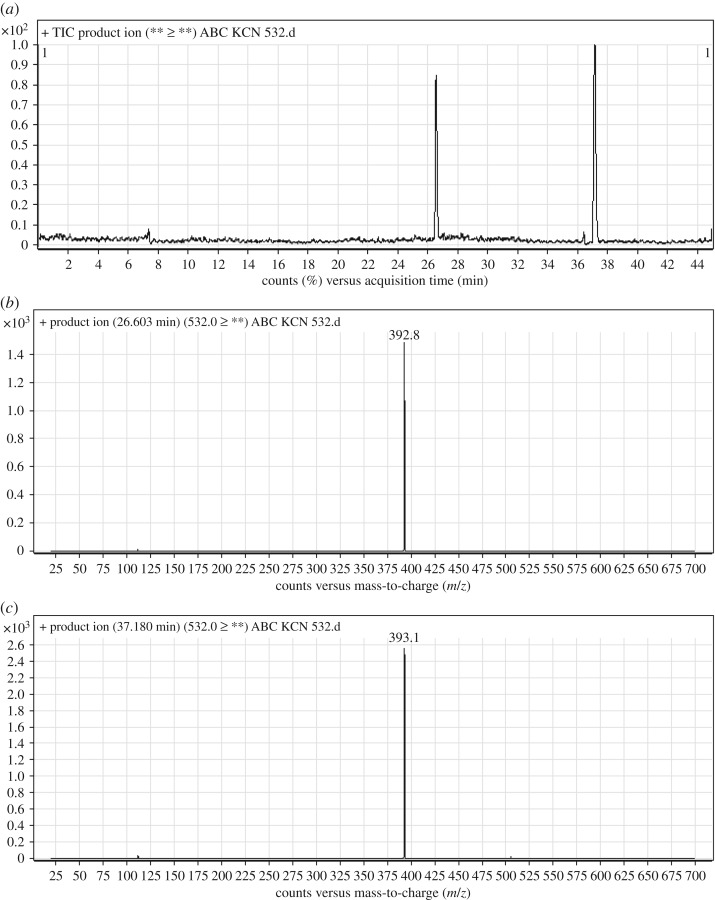
ABC532 PI chromatogram (*a*), ABC532a PI MS spectrum (*b*) and ABC532b PI MS spectrum (*c*).

**Scheme 8.**
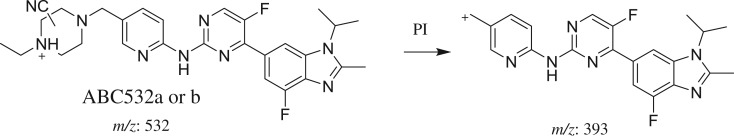
PIs of ABC532a and ABC532b.

ABC532a and ABC532b chromatographic peaks appeared at 26.8 and 37.2 min, respectively, in PI chromatogram (figure 9*a*). CID of ABC532 metabolites generates one FI at *m/z* 393 (figure 9*b*,*c*). Compared with PIs of ABC, PI at *m/z* 393 indicates that all metabolic changes occurred in *N*-ethyl piperazine ring. An increase of 25 *m/z* units indicates that cyano addition occurred in *N*-ethyl piperazine ring (scheme 8).

#### 3.2.8. Identification of ABC548 cyano adduct of ABC

**Figure 10.**
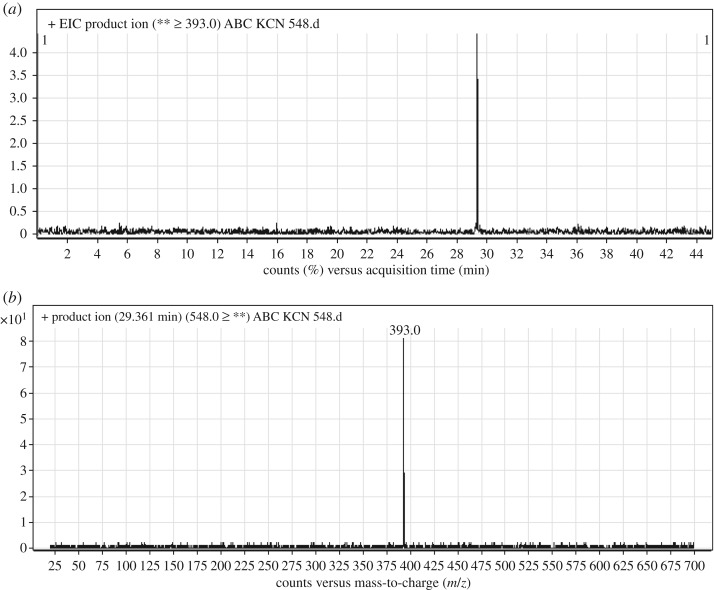
ABC548 PI chromatogram (*a*) and ABC548 PI MS spectrum (*b*).

**Scheme 9.**
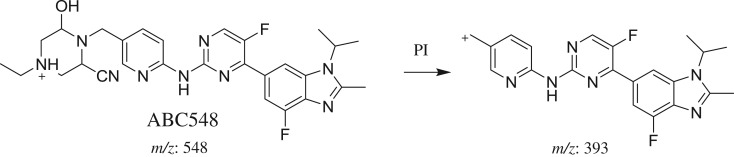
PIs of ABC548.

ABC548 chromatographic peak appears at 29.4 min in PI chromatogram (figure 10*a*). CID of ABC548 generates one FI at *m/z* 393 (figure 10*b*). Compared with PIs of ABC, PI at *m/z* 393 indicates that all metabolic changes occurred in *N*-ethyl piperazine ring. An increase of 41 *m/z* units indicates that hydroxylation and cyano addition occurred in *N*-ethyl piperazine ring (scheme 9).

### 3.3. Supposed pathways of bioactivation of ABC

**Scheme 10.**
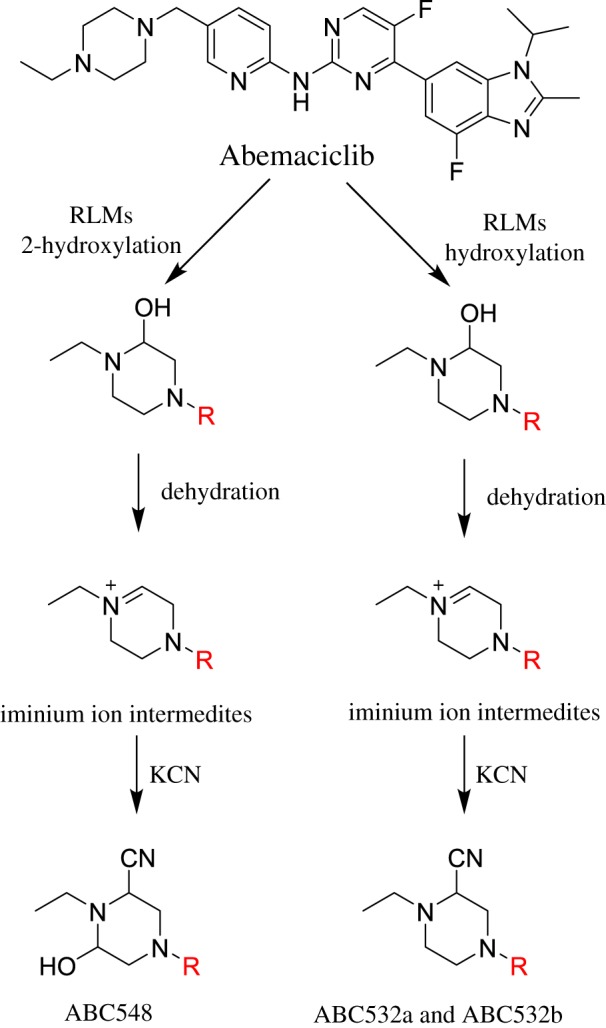
Proposed pathways for the iminium intermediate generation and trapping strategy.

Scheme 10 shows the different pathways for bioactivation of ABC. The formation of ABC532a, ABC532b and ABC548 cyanide adducts confirmed the formation of iminium intermediates in piperazine ring metabolism. Hydroxylation of piperazine ring in ABC followed by dehydration resulted in the generation of iminium ion intermediates that are unstable and reactive that can be trapped by cyanide-forming stable adduct which can be detected in LC–MS/MS. The formation pathway of iminium intermediate and bioactivation mechanism of ABC was previously described with cyclic tertiary amine-containing drugs [10,11,17,20].

## 4. Conclusion

**Figure 11.**
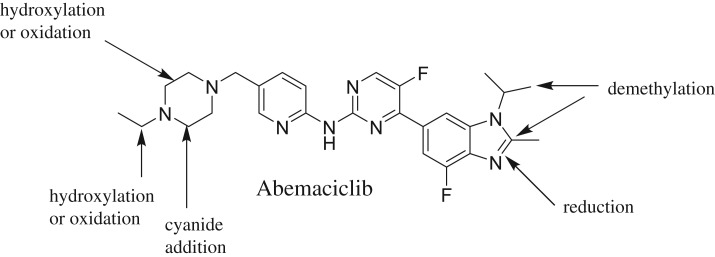
*In vitro* metabolic pathways and bioactivation centres of ABC.

Three potential iminium reactive metabolites were detected and the bioactivation pathways were proposed (figure 11). Nine *in vitro* phase I metabolites were identified. The findings of potentially reactive intermediates of these drugs may give a deeper understanding of their adverse effects. Further drug discovery studies in ABC structure can shed more light on the possibility of blocking or reducing the formation of reactive intermediates by introducing alkyl substituents or isosteric replacement to the alpha position of the piperazine partial moiety which would probably block or interrupt enzymatic oxidation/hydroxylation on α-carbon atoms. This study opens the way for new drug development with more safety profile.

## Supplementary Material

ESM file 1

## Supplementary Material

ESM file 2

## Supplementary Material

ESM file 3

## Supplementary Material

ESM file 4

## Supplementary Material

ESM file 5

## Supplementary Material

ESM file 6

## Supplementary Material

ESM file 7

## Supplementary Material

ESM file 8

## Supplementary Material

ESM file 9

## Supplementary Material

ESM file 10

## Supplementary Material

ESM file 11

## Supplementary Material

ESM file 12

## Supplementary Material

ESM file 13

## Supplementary Material

ESM file 14

## Supplementary Material

ESM file 15

## Supplementary Material

ESM file 16

## Supplementary Material

ESM file 17

## Supplementary Material

ESM file 18

## Supplementary Material

ESM file 19

## Supplementary Material

ESM file 20

## Supplementary Material

ESM file 21

## Supplementary Material

ESM file 22

## Data accessibility

The data supporting the results in this article can be accessed through Dryad Digital Repository at: http://dx.doi.org/10.5061/dryad.71f1s18 [20].
